# Influence of Pregnancy on Whole-Transcriptome Sequencing in the Mammary Gland of Kazakh Mares

**DOI:** 10.3390/ani15142056

**Published:** 2025-07-11

**Authors:** Zhenyu Zhang, Zhixin Lu, Xinkui Yao, Linling Li, Jun Meng, Jianwen Wang, Yaqi Zeng, Wanlu Ren

**Affiliations:** 1 College of Animal Science, Xinjiang Agricultural University, Urumqi 830052, China; 18097907835@163.com (Z.Z.); 18199821647@163.com (Z.L.); yaoxinkui@xjau.edu.cn (X.Y.); lilinling@xjau.edu.cn (L.L.); junm86@sina.com (J.M.); wjw1262022@126.com (J.W.); xjauzengyaqi@163.com (Y.Z.); 2 Xinjiang Key Laboratory of Equine Breeding and Exercise Physiology, Urumqi 830052, China

**Keywords:** horse, equine, mammogenesis, lactogenesis, gestation, whole-transcriptome sequencing

## Abstract

Kazakh mares have drawn significant attention for their outstanding lactation traits. Lactation, a complex physiological activity, is modulated by multiple factors. This study utilized high-throughput sequencing to conduct whole-transcriptome sequencing analysis on the mammary gland tissue of eight Kazakh mares, of which four were pregnant and four were non-pregnant, to systematically reveal the molecular regulatory mechanisms. The results showed differential expression in 2136 mRNAs, 180 lncRNAs, 104 miRNAs, and 1162 circRNAs. Gene ontology functional annotation indicates that these differentially expressed genes are involved in multiple key biological processes, such as the cellular process (BP), metabolic process, and biological regulation. Kyoto Encyclopedia of Genes and Genomes analysis suggests that the differentially expressed genes are significantly enriched in essential pathways such as cytokine–cytokine receptor interaction, the chemokine signaling pathway, and the PI3K-Akt signaling pathway. Additionally, this study constructed a competing endogenous RNA (ceRNA) regulatory network based on the differentially expressed genes (|log_2_FC| > 1, FDR < 0.05), offering a novel perspective for revealing the functional regulation of the mammary gland. This study compared genomic differences in mammary gland tissue of pregnant and non-pregnant Kazakh mares and identified candidate genes that are closely related to lactation regulation. It found that various genes, such as *PIK3CG*, *IL7R*, and *SOD2*, play central regulatory roles in activating mammary gland functions. These findings provide theoretical support for explaining the molecular mechanisms underlying the mammary gland development of Kazakh mares.

## Simple Summary

This study investigated the molecular mechanisms regulating lactation in Kazakh mares, known for their excellent milk production. Using whole-transcriptome sequencing, we compared mammary gland tissue from four pregnant and four non-pregnant mares. The results revealed 2136 differentially expressed mRNAs (e.g., *PIK3CG*, *CLEC4E*), 180 lncRNAs, 104 miRNAs, and 1162 circRNAs. Functional analysis showed that these genes participate in key processes like cellular metabolism and biological regulation, with enrichment in critical pathways such as cytokine–cytokine receptor interaction and PI3K-Akt signaling. Notably, *PIK3CG*, *IL7R*, and *SOD2* were identified as central regulators of mammary gland activation. These findings enhance our understanding of lactation biology in Kazakh mares, providing potential genetic markers for improving milk production in this breed. The study offers valuable insights for equine breeding programs and dairy industry applications.

## 1. Introduction

Kazakh mares are extensively raised due to their remarkable coarse feeding tolerance and stress resistance. This species holds significant economic value as an essential part of the germplasm resources of Equus animals. In Kazakh-mare-rearing regions, herders have long maintained the tradition of drinking horse milk, a practice further promoted by the breeding of dairy horses [1,2,3]. Research indicated that certain mares exhibit persistent lactation during pregnancy, which continues throughout the pregnancy or for extended periods [4]. During pregnancy, mammary gland tissue initially develops ductal systems and forms lobular units with lactation functionality. The growth and development of mammary acini during this period directly influence the number and productivity of mammary epithelial cells during lactation. Upregulation of progesterone and prolactin enhances cell proliferation and mammary ductal branching, thus promoting rapid growth of mammary acini and providing a physiological foundation for subsequent lactation [5].

The mammary gland, a specialized organ unique to mammals, produces milk to nourish offspring. The lactation capability of mares is primarily determined by two factors: the degree of mammary gland development and species-specific characteristics. Mammary gland development is regulated by both genetic and environmental factors. For example, hormones, coding genes, and non-coding RNAs all participate in the modulation of mammary gland cell growth and function. Additionally, other factors such as nutrients, feeding management, and milking frequency also play crucial roles [6]. Anantamongkol et al. [7] investigated the transcriptome of rat mammary gland tissue during pregnancy and lactation, revealing a complex expression mode of transporter genes. Their study indicated that during pregnancy and lactation, multiple transporter genes showed significant alterations, including those involved in the transport of multiple ions and water molecules.

Whole-transcriptome refers to the complete set of transcripts present in a particular type of cell or tissue under specific conditions [8], revealing essential biological regulatory mechanisms. Sevda et al. [9] utilized whole-transcriptome technology to identify genes associated with milk production traits in cows. They found that several genes were essential for milk production across multiple tissue types. Specifically, *LYNX1* is related to milk production traits in eight tissue types, while *DGAT1*, *C14H8orf33*, and *LY6E* are associated with six, five, and five tissue types, respectively. Xia et al. [10] applied the transcriptome technology to analyze changes in the mammary tissue of lactating yaks, identifying over 6000 genes with differential expression, including *BTA3*, *BTA4*, *BTA6*, *BTA9*, *BTA14*, and *BTA28*. Their findings suggested that triacylglycerol synthesis increases during lactation, possibly regulated through the PPAR signal. Among the identified genes, *DGAT1* is critical for milk fat synthesis; *LY6E* regulates mammary stem cell proliferation via the JAK-STAT signaling pathway and plays a key role in mammary gland development; and the *BTA28* chromosomal region in cattle harbors QTL loci associated with lactation traits [11]. This study further reveals the dynamic expression changes in these and related genes during mammary gland development in equine pregnancy through whole-transcriptome analysis. Currently, the application of whole-transcriptome analysis in equine species remains limited. Ren et al. [12] conducted a preliminary investigation of differentially expressed mRNAs in the mammary glands of Kazakh mares during early pregnancy, while Galio et al. [13] reported that *miR-200* inhibits mammary gland development in mice. Although relevant to mammary gland biology, neither study employed a whole-transcriptome approach. Therefore, this present study aims to identify differentially expressed genes in the mammary glands of Kazakh mares during mid-pregnancy using whole-transcriptome sequencing, thereby providing a theoretical foundation and data for future research on equine mammary gland development.

## 2. Materials and Methods

### 2.1. Experimental Animal

A total of 8 healthy Kazakh mares aged between 5 and 8 years were provided by Emin County Wantong Livestock Co. (Emin County, Xinjiang Uygur Autonomous Region, China). All pregnant mares (2–3 parities) were in mid-gestation and nonlactating, with an average gestational age of 150 ± 7 days and a mean body weight of 317.09 ± 25.80 kg. Among them, 4 non-pregnant mares served as the control group (non-pregnant group N), and 4 mid-pregnant mares served as the treatment group (pregnant group P). During the experiment, all mares were housed in separate compartments within the same enclosure and were provided with high-quality dried alfalfa, corn grain, and adequate drinking water. Breast tissue samples were collected using surgical procedures. A sterile, single-use scalpel was employed to excise a tissue section measuring approximately 1 × 0.5 × 0.5 cm from the base of the mammary gland. General anesthesia was induced via intramuscular injection of xylazole (trade name: Jingsongling) at a dosage of 5 mL per 100 kg of body weight. Additionally, 8 mL of procaine hydrochloride was administered for local anesthesia. Following tissue collection, appropriate measures were taken to ensure the horse’s safe recovery from anesthesia, including thorough disinfection, postoperative care, and rehabilitation of the surgical site. Environmental conditions during sampling were recorded as follows: on the first day, the average ambient temperature was −3.0 ± 3.0 °C (range: −6 to 0 °C), with a constant relative humidity of 35%. On the second day, the average temperature was −5.0 ± 5.0 °C (range: −10 to 0 °C), and the relative humidity remained stable at 68%. Mammary gland tissue samples were collected once in November 2024 and immediately preserved in liquid nitrogen for subsequent analyses.

### 2.2. Transcriptome Sequencing

Total RNAs were extracted from mammary gland tissues, and the mRNAs were enriched using mRNA capture magnetic beads. For cDNA library construction, we employed HieffNGS^®^ DNA Selection Beads (Yeasen Biotechnology, Shanghai, China), a SPRI-based magnetic bead system optimized for Illumina platform compatibility. The enriched mRNAs were subsequently fragmented and used for cDNA synthesis. Following cDNA purification with Hieff NGS^®^ DNA Selection Beads, target fragments were screened, and PCR library amplification was conducted to establish libraries. High-throughput sequencing was performed on the Illumina Novaseq X Plus platform. All procedures were carried out by Guangzhou Gene Denovo Biotechnology Co. (Guangzhou, China).

### 2.3. Data Quality Control

To ensure high data quality, quality control steps were implemented before statistical analysis. Adapter sequences, repetitive sequences, and low-quality reads were removed using fastp [14], resulting in high-quality clean reads [15], thereby minimizing the impact of invalid data on subsequent analyses.

### 2.4. Relationship Analysis of Samples

PCA was performed using R software 4.3.1 based on gene expression data. By employing a dimensionality reduction approach, the distance relationships among samples were analyzed to evaluate differences in expression patterns and intra-group consistency across Group N and Group P. Pearson correlation analysis was performed to evaluate gene expression relationships among samples, and the resulting correlation coefficients were visualized using a heatmap to illustrate the pairwise correlations between samples.

### 2.5. Differential Expression Analysis

Expression quantification of mRNAs, lncRNAs, miRNAs, and circRNAs was performed using three standardized metrics: reads per million (RPM), fragments per kilobase of transcript per million mapped reads (FPKM), and transcripts per million (TPM). Differential expression analysis was conducted using DESeq2 R package 1.48.1 [16], which employs a negative binomial distribution model tailored for RNA-seq count data. To account for multiple hypothesis testing, the Benjamini–Hochberg (BH) method was applied to control the false discovery rate. Differentially expressed mRNAs and lncRNAs were identified based on the criteria of |log_2_FC| > 1 and FDR < 0.05, while miRNAs and circRNAs were considered differentially expressed if they exhibited a fold change ≥ 2 and a *p*-value ≤ 0.05.

### 2.6. Gene Ontology (GO) and Kyoto Encyclopedia of Genes and Genomes (KEGG) Enrichment Analysis

Functional annotation analysis of differentially expressed mRNAs and their host non-coding RNAs was carried out using the GO and KEGG databases. GO functional classification was performed using the Goseq method to correct for gene length bias. KEGG pathway enrichment analysis was conducted using KOBAS software 3.0 to assess the enrichment of differentially expressed genes within specific pathways. The calculated *p*-values were subjected to FDR Correction, taking FDR ≤ 0.05 as a threshold.

### 2.7. RT-qPCR Validation

The HiScript^®^ Q RT SuperMix for RT-qPCR assay kit (Vazyme, Nanjing, China) was used to reverse transcribe total RNA into cDNA. To validate the transcriptome sequencing results, 10 mRNAs were randomly selected for quantitative real-time PCR analysis. RT-qPCR primers were designed using the software Primer 5, which was based on genetic sequence information from the primer synthesis department. RT-qPCR was performed using the ChamQ SYBR RT-qPCR Master Mix assay kit (Vazyme), with GAPDH and β-actin serving as internal reference genes.

### 2.8. Statistical Analysis

Statistical analysis, including one-way analysis of variance (ANOVA) and multiple comparison tests, was performed using SPSS 26.0 to compare mammary gland tissues between Group P and Group N.

## 3. Results

### 3.1. Expression of mRNAs, lncRNAs, miRNAs, and circRNAs in the Samples

**Figure 1:**
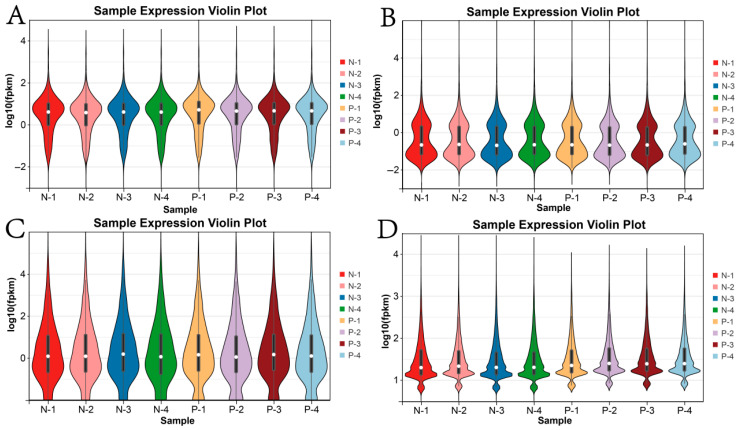
Violin plots illustrating gene expression. (**A**) mRNAs; (**B**) lncRNAs; (**C**) miRNAs; (**D**) circRNAs. Note. N-1 to N-4 represent the four mares in the non-pregnant group (Group N), while P-1 to P-4 represent the four mares in the pregnant group (Group P). The same nomenclature applies throughout.

A certain degree of variation in mRNA expression was observed among the different samples, where the heterogeneity of gene expression in Group P was slightly higher than that in Group N. This may be attributed to the biological properties of the samples, as additional regulatory factors can influence gene expression in Group P (Figure 1A). In contrast, miRNAs and circRNAs exhibited less variation in gene expression across the samples, indicating a more consistent expression pattern between samples from Groups N and P (Figure 1B–D).

### 3.2. Correlation and Clustering Analysis of mRNAs, lncRNAs, miRNAs, and circRNAs

**Figure 2:**
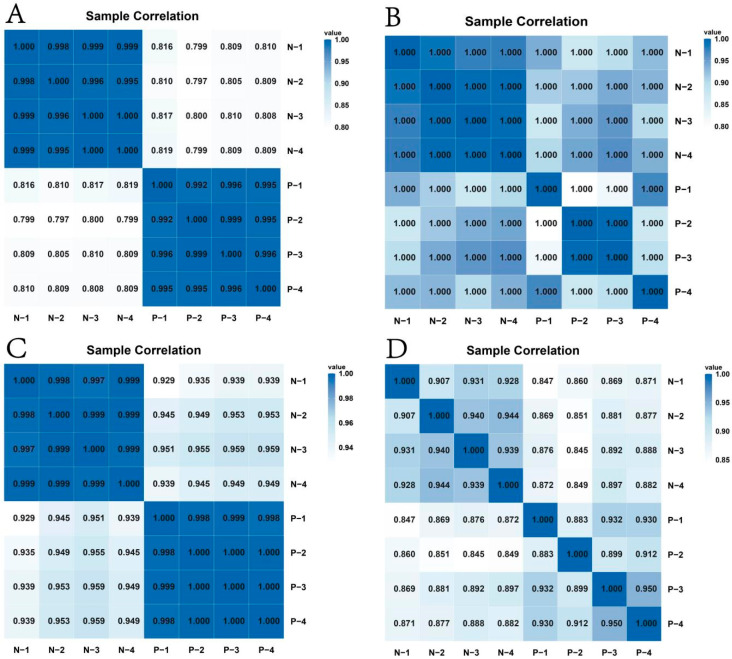
Heatmap showing the correlation among samples. (**A**) mRNAs; (**B**) lncRNAs; (**C**) miRNAs; (**D**) circRNAs.

The heatmaps of mRNAs (Figure 2A) and lncRNAs (Figure 2B) revealed higher correlation and relatively unified expression patterns among the samples. Conversely, miRNAs (Figure 2C) and circRNAs (Figure 2D) exhibited lower correlation coefficients, suggesting greater expression heterogeneity. Specifically, with mRNAs and lncRNAs showing stable expression patterns across samples, circRNAs demonstrated more pronounced expression differences, potentially reflecting sample-specific biological properties.

### 3.3. PCA of mRNAs, lncRNAs, miRNAs, and circRNAs

**Figure 3:**
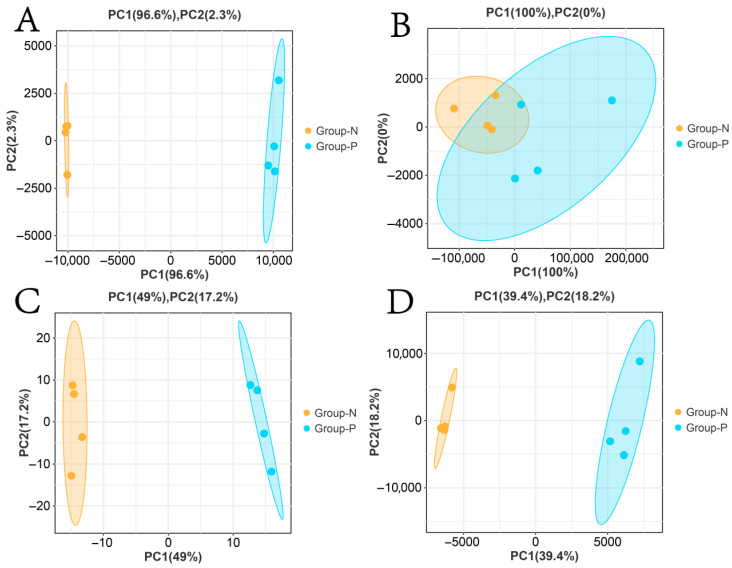
PCA clustering of Group N and Group P samples. (**A**) mRNAs; (**B**) lncRNAs; (**C**) miRNAs; (**D**) circRNAs.

PCA results demonstrated significant separation in the principal component space for mRNAs, lncRNAs, miRNAs, and circRNAs (Figure 3A,C,D). For lncRNAs, the first principal component accounted for 100% of the variance. Although overlap was observed in the distribution areas between Groups N and P, Group P samples exhibited a broader distribution along the PC1 axis, whereas Group N was more tightly clustered. This indicated the difference in lncRNA expression between the groups, with a less pronounced separation compared to mRNAs, miRNAs, and circRNAs (Figure 3B).

### 3.4. Analysis of Differential Expression of mRNAs, lncRNAs, miRNAs, and circRNAs

**Figure 4:**
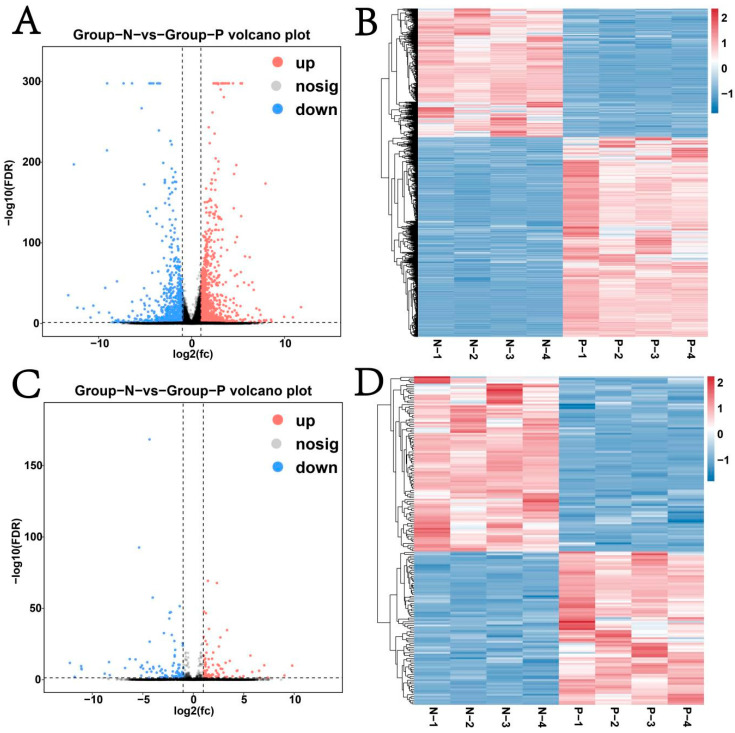
Differential expression analysis of mRNAs and lncRNAs. (**A**) Volcano plot of differentially expressed mRNAs; (**B**) heat map of differentially expressed mRNAs; (**C**) volcano plot of differentially expressed lncRNAs; (**D**) heat map of differentially expressed lncRNAs.

The volcano plot results showed that both mRNAs and lncRNAs had differentially expressed genes (Figure 4A,C). Among them, differential expression analysis identified 1300 significantly upregulated (*p* ≤ 0.05) mRNAs, including *PIK3CG*, *CLEC4E*, *UBD*, and *SOD2* and 836 remarkably downregulated mRNAs, including *H1-5*, *GUCY1B1*, and *C11H17* (Figure S1A). Similarly, 84 lncRNAs were obviously upregulated (*p* ≤ 0.05), and 96 were downregulated (Figure S1B). Heatmaps clearly differentiated the expression patterns of mRNAs and lncRNAs between Groups N and P (Figure 4B,D).

**Figure 5:**
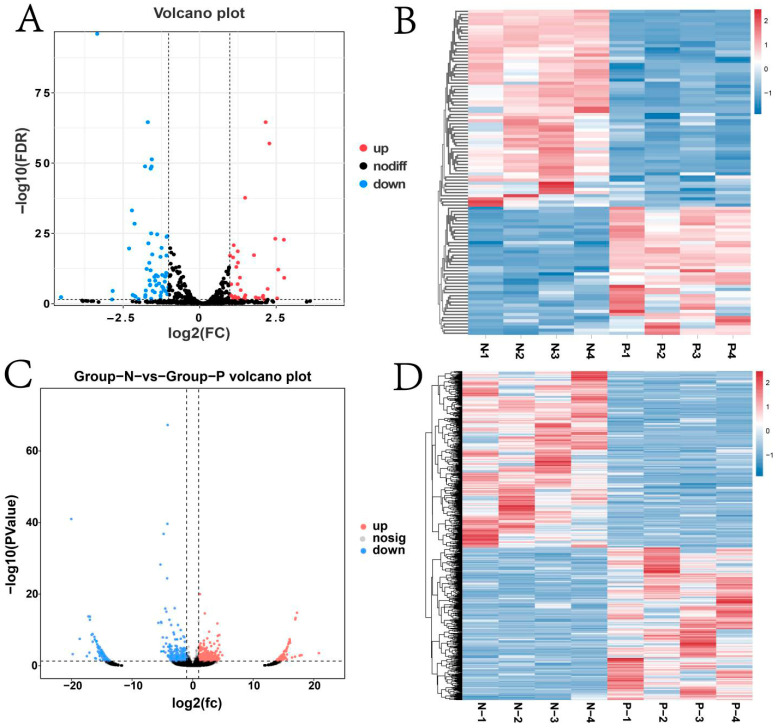
Differential expression analysis of miRNA and circRNA (**A**) Volcano plot of differentially expressed miRNAs; (**B**) heat map of differentially expressed miRNAs; (**C**) volcano plot of differentially expressed circRNAs; (**D**) heat map of differentially expressed circRNAs.

The volcano plot results showed that both miRNAs and circRNAs had differentially expressed genes (Figure 5A,C). For miRNAs, 41 were significantly upregulated (*p* ≤ 0.05), including *eca-miR-1-146a*, *eca-miR-122*, and *eca-miR-21*, and 63 were significantly downregulated (*p* ≤ 0.05), such as miR-375-y, eca-miR-9a, and eca-miR-411 (Figure S1C). Regarding circRNAs, 539 were significantly upregulated (*p* ≤ 0.05) such as *novel_circ_025824*, *novel_circ_031697*, and *novel_circ_009239*, and 623 were significantly downregulated (*p* ≤ 0.05) including *novel_ circ_021617*, *novel_circ_015998*, and *novel_circ_021619*. (Figure S1D). Notably, distinct expression patterns of circRNAs were observed between the two groups (Figure 5B,D).

### 3.5. GO and KEGG Enrichment Analysis

**Figure 6:**
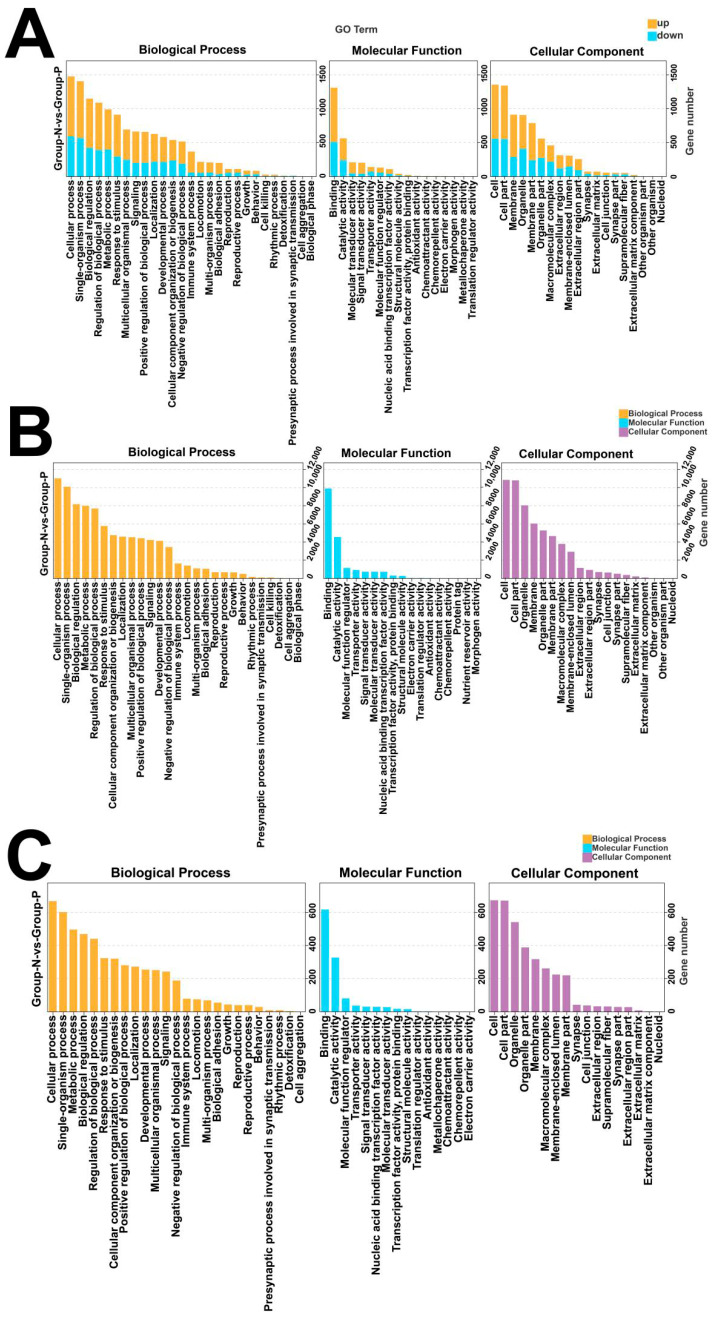
GO enrichment of differentially expressed mRNAs, lncRNAs, and circRNAs. (**A**) GO enrichment of mRNAs; (**B**) GO enrichment of miRNA target genes; (**C**) GO enrichment of source genes of circRNAs.

GO enrichment analysis indicated that differentially expressed mRNAs are primarily enriched in pathways including the cellular process (BP), metabolic process, and biological regulation (Figure 6A). Similarly, differentially expressed miRNAs and circRNAs were also majorly enriched in these categories (Figure 6B,C).

**Figure 7:**
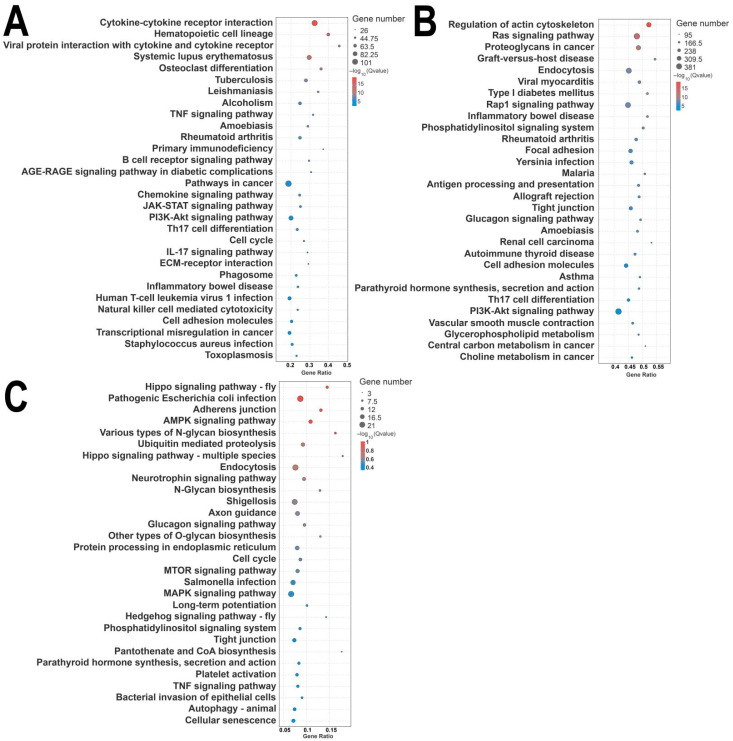
KEGG enrichment of differentially expressed mRNAs, lncRNAs, and circRNAs. (**A**) KEGG enrichment of mRNAs; (**B**) KEGG enrichment of LncRNA target genes; (**C**) KEGG enrichment of source genes of circRNAs. Note: GO enrichment results are illustrated in categorized histograms with the second GO item on the x-axis, and DEG counts on the y-axis. Red bars indicate upregulation, and green bars indicate downregulation. KEGG enrichment is shown as a bubble chart, highlighting the 20 pathways with the lowest Q-values. The y-axis represents pathways, the x-axis represents enrichment factors, the bubble size reflects gene number, and deeper red indicates a lower Q-value.

KEGG pathway enrichment analysis indicated that differentially expressed mRNAs are primarily enriched in pathways such as cytokine–cytokine receptor interaction, hematopoietic cell lineage, chemokine signaling pathway, and PI3K-Akt signaling pathway (Figure 7A). Differentially expressed miRNAs were mainly associated with the MAPK signaling pathway, PI3K-Akt signaling pathway, neuroactive ligand-receptor interaction, and others (Figure 7B), while differentially expressed circRNAs were mainly enriched in the MAPK signaling pathway, the endocrine system, and neuroactive ligand-receptor interaction (Figure 7C).

### 3.6. Gene Interaction Network Analysis

Gene interaction networks were constructed based on differentially expressed RNAs to explore the molecular mechanism underlying mammary gland function during pregnancy in Kazakh mares. Key nodes identified include ko01100, ko05200, and ko04060 in the mRNA interaction network; ko01100, ko05200, and ko04151 in the miRNA interaction network; and ko05130, ko04520, and ko04144 in the circRNAs interaction network. Notably, ko01100 and ko05200 were shared between the mRNA and miRNA networks, suggesting that metabolic and proliferation pathways constitute the core regulatory processes of mammary gland function. The extensive interconnectivity of these nodes highlighted the complexity of the coregulatory mechanisms involved (Table 1). Further details are provided in Supplementary Tables S1–S3.

### 3.7. Association Analyses of mRNAs and lncRNAs

**Figure 8:**
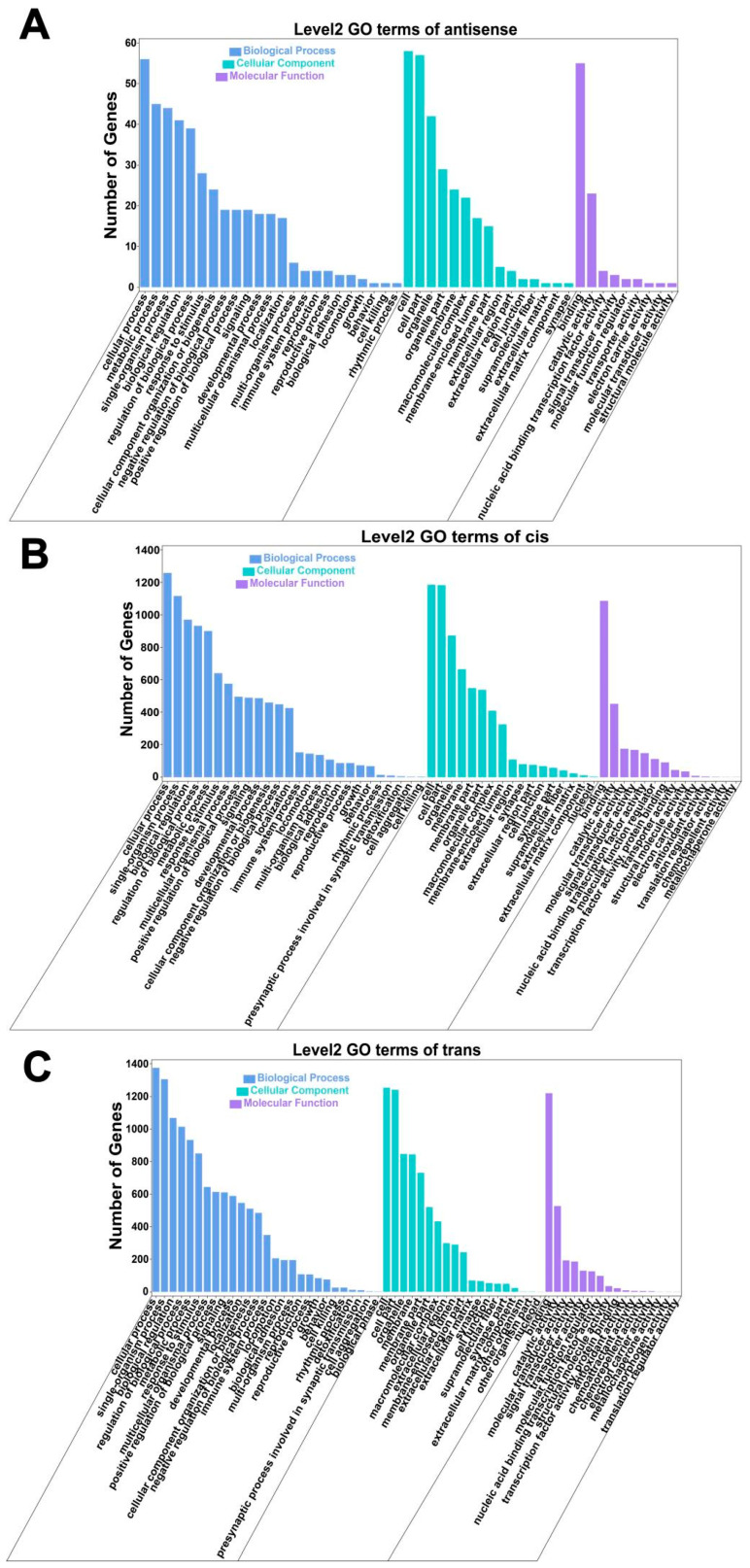
GO enrichment classification of antisense-related and cis/trans-regulated genes. (**A**) Histogram of antisense-associated GO enrichment categories; (**B**) Histogram of cis-regulated GO enrichment classification; (**C**) Histogram of trans-regulated GO enrichment classification.

**Figure 9:**
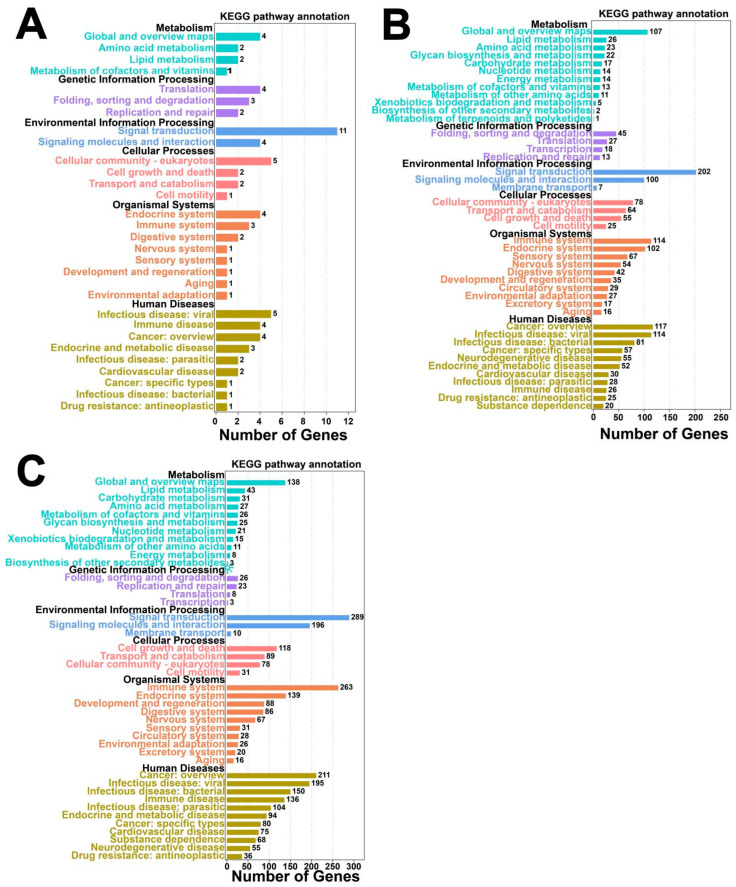
KEGG pathway enrichment analysis of antisense-associated and cis/trans-regulated genes. (**A**) Bar chart of antisense-associated KEGG pathway enrichment; (**B**) Bar chart of cis-regulatory KEGG pathway enrichment; (**C**) Bar chart of trans-regulatory KEGG pathway enrichment.

The GO biological processes of antisense-associated genes are primarily enriched in categories such as regulation of biological processes, response to stimulus, metabolic processes, cellular processes (BP), and developmental processes (Figure 8A). KEGG pathway enrichment analysis indicated significant involvement in global and overview maps, folding, sorting, degradation, signal transduction, the endocrine system, the immune system, and other pathways (Figure 9A).

### 3.8. Cis-Regulatory Elements Analysis

The GO biological processes of cis-regulatory genes are predominantly enriched in the cellular process (BP), metabolic process, and biological regulation (Figure 8B). Similarly, KEGG pathway analysis revealed enrichment in global and overview maps, folding, sorting, degradation, signal transduction, the endocrine system, and the immune system (Figure 9B).

### 3.9. Trans-Regulatory Elements Analysis

The GO biological processes associated with trans-regulation elements were mainly enriched in the cellular process (BP), metabolic process, biological regulation, and others (Figure 8C). KEGG enrichment pathways analysis identified significant involvement in global and overview maps, lipid metabolism, carbohydrate metabolism, amino acid metabolism, and metabolism of cofactors and vitamins, among other pathways (Figure 9C).

### 3.10. ceRNA Analysis

**Figure 10:**
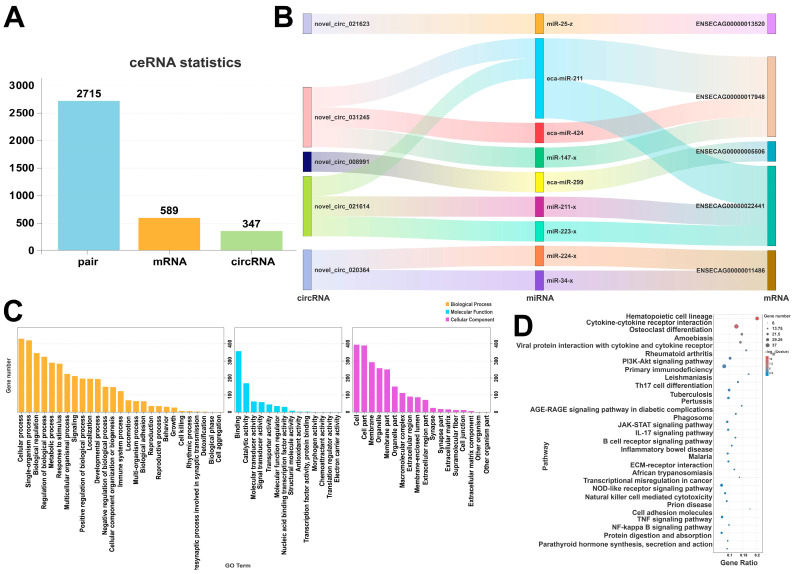
(**A**) Statistical plot of interactive ceRNAs, (**B**) Sankey diagram of interactive ceRNAs, (**C**) GO enrichment plot of interactive ceRNAs, (**D**) KEGG enrichment plot of interactive ceRNAs.

By integrating transcriptome sequencing data, a ceRNA regulatory network for Kazakh mares was analyzed, along with the competing regulatory relationship between circRNAs, miRNAs, and mRNAs. The results are shown in Figure 10A, where 2715 corresponding regulatory pairs were identified with 589 mRNAs and 347 circRNAs. Specifically, Figure 10B shows that multiple circRNAs, such as *novel_circ_021623* and *novel_circ_031245*, competitively bind to miRNAs, including *ca-miR-211* and *eca-miR-424*, thus regulating the expression of mRNAs such as *ENSECAG00000017948* and *ENSECAG00000001486*. Furthermore, *miR-147-x* and *miR-224-x* were found to interact with multiple circRNAs and mRNAs. Additionally, mRNAs such as *ENSECAG00000022441* and *ENSECAG00000005506* were under the coregulation of various miRNAs.

Figure 10C demonstrates the GO enrichment analysis. It can be observed that the mRNAs within the ceRNA network were significantly enriched in categories such as the single-organism process, cellular processes (BP), and biological regulation (*p* ≤ 0.05). KEGG enrichment analysis (Figure 10D) shows that mRNAs associated with the ceRNA network were significantly enriched in pathways related to reproduction and metabolism (*p* ≤ 0.05), including the PI3K-Akt signaling pathway, TGF-β signaling pathway, and cytokine–cytokine receptor interaction. In addition, the mRNAs were also enriched in the AGE-RAGE signaling pathway and ECM-receptor interaction pathways.

### 3.11. RT-qPCR Analysis

**Figure 11:**
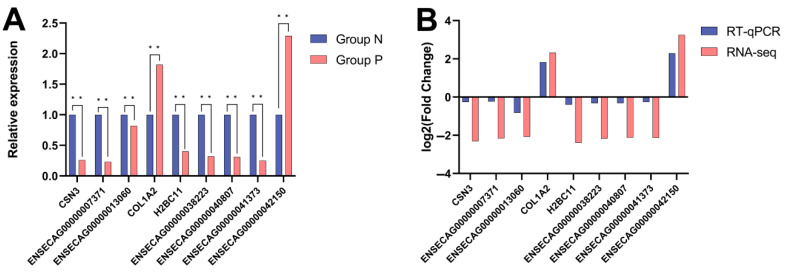
Validation of differentially expressed genes by RT-qPCR. (**A**) Log_2_ fold-change comparison between RNA-seq and RT-qPCR for differentially expressed genes; (**B**) Relative expression of differentially expressed genes by RT-qPCR. Note: In the figure, * * represents extremely significant.

To validate the reliability of RNA-seq data, 10 differentially expressed mRNAs were randomly selected for RT-qPCR analysis (Supplementary Table S4). The results revealed that the expression trends observed in RNA-seq and RT-qPCR were consistent, thereby confirming the credibility of the RNA-seq data (Figure 11A,B).

## 4. Discussion

Mammary gland development in mammals can be divided into six stages: embryonic, juvenile, pubertal, pregnancy, lactation, and involution [17]. Progesterone acts at the beginning and the end, while estrogens and progestagens act in between, continuously stimulating the acinar cells over several months to support mammogenesis during mare pregnancy [18,19]. At this stage, the lobules of the mammary gland experience an increase in the number and size of acini, exhibiting substantial growth. Meanwhile, the acini undergo an active state, reaching a quantity during pregnancy. Toward the end of pregnancy, under the influence of progesterone and prolactin, the acini begin to produce milk in preparation for the subsequent lactation period [20]. Therefore, the development of the mammary gland during pregnancy directly determines both the quantity of acini and their lactation [21,22]. Ren et al. [1] identified *WNT4*, *DPP4*, and *NFKBIA* as key regulatory nodes involved in the activation of the mammary gland. In the present study, genes differentially expressed between pregnant and non-pregnant mares were primarily enriched in the NF-κB signaling pathway, steroid hormone biosynthesis, ECM-receptor interaction, and the MAPK signaling pathway.

### 4.1. Differential RNA Analysis of the Mammary Gland Across Pregnant and Non-Pregnant Kazakh Mares

The upregulation of *SOD2* may be associated with enhanced resistance of the mammary gland to oxidative stress during pregnancy. *SOD2*, a mitochondrial antioxidant enzyme, converts superoxide radicals into oxygen and hydrogen peroxide, thus mitigating oxidative damage. In mammary gland tissue, *SOD2* expression influences cell development, differentiation, and lactation, all of which are essential in managing oxidative stress [23]. Sun et al. [24] reported that in bovine mammary cells, high *SOD2* expression helps alleviate oxidative damage to mammary epithelial cells. The high metabolic demand of the mammary gland during pregnancy may generate an increased level of reactive oxygen species, making *SOD2* upregulation critical for protecting mammary epithelial cells and supporting subsequent lactation. Similarly, Zhang et al. [25] found that the elevated *IL7R* expression is linked to reshaping the tumor immune microenvironment by regulating the activity of γ δ T cells, contributing to immune homeostasis. A similar mechanism may operate in the mammary glands of pregnant mares, supporting immune balance during pregnancy.

This study also revealed that specific miRNAs, such as *eca-miR-21*, influence the proliferation and differentiation of mammary epithelial cells through the PI3K-Akt and MAPK signaling pathways. Lai et al. [26] identified in bovine mammary glands that *bta-miR-21* modulates these pathways, promoting mammary epithelial cell growth in preparation for lactation. circRNAs, a class of stable non-coding RNAs forming closed circular structures, serve as sponges that absorb miRNAs, preventing them from repressing their target genes and thus indirectly upregulating gene expression [27]. Wang et al. [28] demonstrated that circRNAs can sponge *miR-148a/152*, which in turn leads to an upregulation of its target genes that may enhance the proliferation of mammary epithelial cells. Research shows that *miR-148a/152* usually inhibits certain genes that are associated with proliferation, such as *DNMT1*. Therefore, by sponging this miRNA, circRNAs can contribute to the high expression of target genes, which consequently results in enhanced proliferation. This process plays a key role during the development of mammary glands and lactation. Additionally, the upregulation of *eca-miR-146a* may be associated with the immunoregulation and growth of mammary tissues. Studies have reported that in bovine mammary glands, *eca-miR-146a* is involved in regulating mammary ductal development and lactation through the NF-κB signaling pathway, maintaining tissue homeostasis by balancing cell proliferation, differentiation, and apoptosis [29].

### 4.2. Participation of Cytokine–Cytokine Receptor Interaction in Lactation Stimulation

The cytokine–cytokine receptor interaction pathway plays a crucial role in immunoregulation and tissue remodeling in the mammary gland during pregnancy. Watson et al. [30] demonstrated that mammary gland tissues regulate immune responses and inflammation through cytokine signaling, supporting acini formation and preparing for lactation. This pathway involves multiple cytokines and their receptors, modulating innate and adaptive immune responses as well as cellular development and differentiation [31]. This study identified 26 genes in this pathway through KEGG enrichment analysis (*p* ≤ 0.05), highlighting its importance in the regulation of the immune microenvironment. For example, mammary gland tissues during pregnancy must balance the inflammatory response to avoid excessive immune activation. Cytokine pathways regulate T-cell and macrophage activities, thus ensuring normal mammary function [32]. Zhao et al. [33], in a study of porcine mammary transcriptomes during late pregnancy, found significant activation of the cytokine–cytokine receptor interaction pathway on day 110 of gestation compared to days 80 and 100, particularly regulation of *IL-4*, suggesting active immune involvement in the mammary microenvironment. Alluwaimi et al. [34] observed that this pathway promotes immune cell recruitment and tissue remodeling during bovine pregnancy, supporting lactation. Thus, the cytokine–cytokine receptor interaction pathway in pregnant Kazakh mares may similarly mediate immune regulation and tissue development.

### 4.3. Function of the ceRNA Network

Network analysis of ceRNAs showed that *novel_circ_021623* and *novel_circ_031245* regulate the expression of mRNAs such as *ENSECAG00000017948* and *ENSECAG00000005506* by competitively binding to *eca-miR-211* or *eca-miR-424*. mRNAs, including *miR-147-x* and *miR-224-x*, showed high degrees of interaction within the network, suggesting that they may play a key role in mammary gland regulation during pregnancy. Additionally, the PI3K-Akt signaling pathway, TGF-β signaling pathway, and ECM-receptor interaction pathway are closely related to mammary epithelial cell proliferation, extracellular matrix remodeling, and inflammatory response. Zhu et al. [35] found that *circRNA8220* promotes milk fat synthesis by regulating the PI3K-Akt-mTOR pathway in the mammary gland of cows, which is consistent with our findings. Activation of the TGF-β pathway enhances the formation of mammary lobular-acini structures by regulating mammary epithelial cell differentiation, aligning with findings by Wakefield L.M. et al. [36] in mice, suggesting that this phenomenon is widespread and highly conserved in mammals. The role of the TGF-β signaling pathway in mammary gland development has been confirmed by Brisken et al. [37], indicating that it promotes acini formation by regulating cell proliferation and differentiation. Mu et al. [38] reported that ceRNA networks influence the adhesion and migration of mammary epithelial cells in cows through the regulation of the ECM-receptor interaction pathway, which suggests that the ceRNA network is closely related to the structural development of mammary tissues. The regulation of the TGF-β pathway by the network of ceRNAs in the present study further demonstrated that the mammary gland during pregnancy may promote an increase in the number of acini and lactation through the mechanism of ceRNA interactions.

Several candidate molecules identified, including *PIK3CG*, *eca-miR-21*, and *novel_circ_025824*, are associated with lactation regulation, offering theoretical support for the genetic improvement of lactation performance in Kazakh mares. Meanwhile, the integration of proteomics and metabolomics analyses will further reveal the molecular regulatory mechanisms of the mammary gland during gestation. The potential interaction between circRNAs and hormone signaling is another area of interest. Chen et al. [39] found that circRNAs influence cell differentiation by modulating hormone-signaling-related genes, a mechanism that might also operate in Kazakh mares. Epigenetic regulation likely constitutes another crucial layer in mammary gland development. Notably, certain differentially expressed genes, such as *eca-miR-146a*, may contribute to mastitis resistance, and their high expression may reduce the risk of disease by suppressing the inflammatory response. This was also demonstrated by Lawless et al. [40] in bovine mammary glands. Bach et al. [41] have unveiled dynamic gene expression profiles and the underlying regulatory mechanisms of adipocyte and epithelial cells during differentiation, proliferation, and function formation in the mammary glands of mice through single-cell transcriptomic sequencing. Ostrander et al. [42] incorporated mammary gland developmental data to uncover the genetic basis of lactation performance in cows and sheep through comparative genomics analysis. Future research employing single-cell transcriptome sequencing and cross-species genome comparisons could further explore the genetic underpinnings of pregnancy and lactation in Equus species, providing a solid foundation for breeding strategies aimed at optimizing mammary gland function.

This study employed whole-transcriptome sequencing to identify key genes and regulatory networks associated with mammary gland development during pregnancy in Kazakh mares, providing a theoretical foundation for the development of molecular breeding markers, optimization of pregnancy management strategies, and improvement in milk production performance. Future research will focus on the cultivation of primary mammary gland cells from Kazakh mares, integration of single-cell transcriptomic sequencing, and functional validation using model organisms. Additionally, we plan to conduct cross-species comparisons and large-scale validation studies to facilitate the translation of fundamental discoveries into practical applications in livestock production. However, several limitations of the present study were acknowledged. All samples were obtained from pregnant mares at a similar mid-gestational stage (150 ± 7 days), making sample collection logistically challenging. Although this conforms to accepted research standards for studies on mammary gland development in large animals, the overall sample size was limited. Furthermore, experimental comparisons were conducted individually, without the formation of pooled consortia. To address these limitations, future studies will include a broader range of samples, employ pooled consortia for comparisons, and establish collaborations with equine breeding centers to increase the sample size, thus enhancing the robustness of the findings.

## 5. Conclusions

Through whole-transcriptome technology, this study systematically compared the mammary gland tissue from Kazakh mares during mid-pregnancy and non-pregnant states. It reveals the molecular mechanism by which pregnancy regulates mammary gland function and lactation preparation. Several DEGs, including *PIK3CG*, *IL7R*, and *SOD2*, were identified as essential players in mid-pregnancy mares that may function through pathways such as the MAPK signaling pathway, the PI3K-Akt signaling pathway, and the neuroactive ligand-receptor interaction. These pathways collectively promote the proliferation and activation of mammary gland cells, laying the foundation for lactation.

## Figures and Tables

**Table 1:** KEGG network interaction of the differentially expressed mRNAs, miRNAs, and circRNAs.

RNA	KEGG_A_Class	KEGG_B_Class	Pathway	Group-N-vs-Group-P	All	*p* Value	Q Value	Pathway ID
mRNAs	Metabolism	Global and overview maps	Metabolic pathways	145	1516	0.995437	1.000000 × 10^+0^	ko01100
	Human Diseases	Cancer: Overview	Pathways in cancer	101	537	1.630413 × 10^−7^	3.586909 × 10^−6^	ko05200
	Environmental Information Processing	Signaling molecules and interaction	Cytokine–cytokine receptor interaction	95	291	7.933928 × 10^−23^	2.618196 × 10^−20^	ko04060
miRNAs	Metabolism	Global and overview maps	Metabolic pathways	1287	3551	0.00065877	3.238181 × 10^−3^	ko01100
	Human Diseases	Cancer: Overview	Pathways in cancer	527	1334	5.577158 × 10^−6^	5.406745 × 10^−5^	ko05200
	Environmental Information Processing	Signal transduction	PI3K-Akt signaling pathway	381	916	4.260542 × 10^−7^	5.718958 × 10^−6^	ko04151
circRNAs	Metabolism	Global and overview maps	Metabolic pathways	72	1513	0.09367813	0.61050942	ko01100
	Human Diseases	Infectious disease: bacterial	Pathogenic Escherichia coli infection	21	241	0.000910662	0.08811273	ko05130
	Cellular Processes	Transport and catabolism	Endocytosis	20	261	0.005352532	0.18711521	ko04144

## Data Availability

The data presented in this study are openly available in BioProject (reference number: PRJNA1256214).

## Supplementary Materials

The following supporting information can be downloaded at: https://www.mdpi.com/article/10.3390/ani15142056/s1, Figure S1: Differential Expression Profiles of mRNAs, lncRNAs, miRNAs, and circRNAs. (A) number of differentially expressed mRNAs; (B) number of differentially expressed lncRNAs; (C) number of differentially expressed miRNAs; (D)number of differentially expressed circRNAs. Table S1: mRNAs KEGG network interaction relationship; Table S2: miRNAs KEGG network interaction relationship; Table S3: circRNAs KEGG network interaction relationship; Table S4: Primer Information Table for RT-qPCR.
