# Supplementary figures and images for: Influence of Pregnancy on Whole-Transcriptome Sequencing in the Mammary Gland of Kazakh Mares
Source: Animals (Basel). 2025 Jul 11;15(14):2056. doi: 10.3390/ani15142056 (PMC12291736; doi:10.3390/ani15142056)

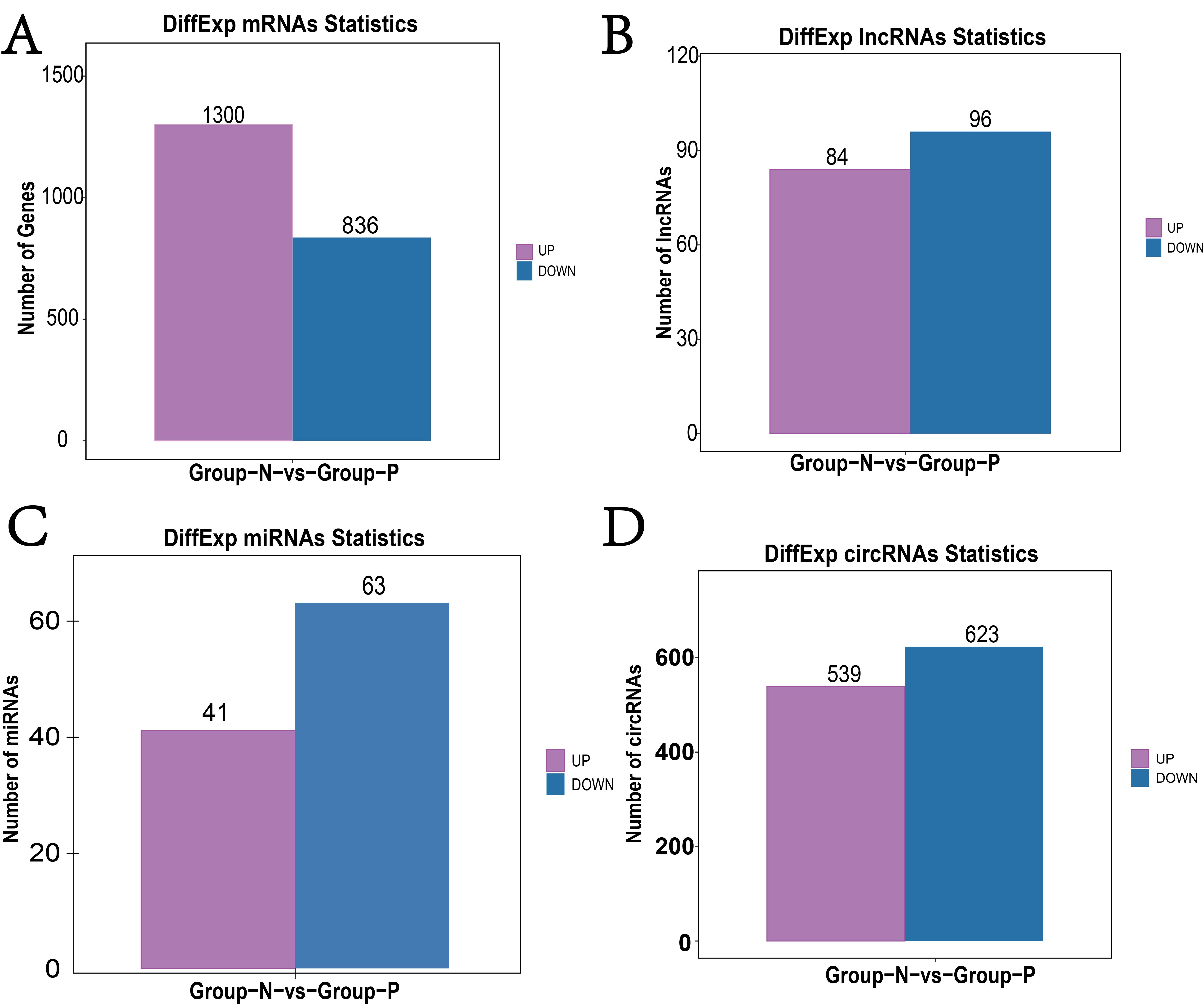

Supplement: Supplementary file 1 [file animals-15-02056-s001.zip › Supplementary Figure S1.jpg]
